# Texture-based markers from structural imaging correlate with motor handicap in Parkinson’s disease

**DOI:** 10.1038/s41598-021-81209-4

**Published:** 2021-02-01

**Authors:** Nacim Betrouni, Caroline Moreau, Anne-Sophie Rolland, Nicolas Carrière, Marie Chupin, Gregory Kuchcinski, Renaud Lopes, Romain Viard, Luc Defebvre, David Devos

**Affiliations:** 1 Université de Lille, INSERM, U1172, CHU-Lille, Lille Neuroscience Cognition Research Centre, 1 place de Verdun, 59000 Lille, France; 2 Neurology and Movement Disorders Department, CHU Lille, Licend, 59000 Lille, France; 3 CATI, Institut du Cerveau et de le Moelle Epinière, INSERM, U1127, CNRS, UMR7225, Sorbonne, Université, 75013 Paris, France; 4 Department of Neuroradiology, CHU Lille, Licend, 59000 Lille, France

**Keywords:** Neuroscience, Biomarkers, Neurology, Engineering

## Abstract

There is a growing need for surrogate biomarkers for Parkinson’s disease (PD). Structural analysis using magnetic resonance imaging with T1-weighted sequences has the potential to quantify histopathological changes. Degeneration is typically measured by the volume and shape of morphological changes. However, these changes appear late in the disease, preventing their use as surrogate markers. We investigated texture changes in 108 individuals, divided into three groups, matched in terms of sex and age: (1) healthy controls (n = 32); (2) patients with early-stage PD (n = 39); and (3) patients with late-stage PD and severe L-dopa-related complications (n = 37). All patients were assessed in off-treatment conditions. Statistical analysis of first- and second-order texture features was conducted in the substantia nigra, striatum, thalamus and sub-thalamic nucleus. Regions of interest volumetry and voxel-based morphometry were performed for comparison. Significantly different texture features were observed between the three populations, with some showing a gradual linear progression between the groups. The volumetric changes in the two PD patient groups were not significantly different. Texture features were significantly associated with clinical scores for motor handicap. These results suggest that texture features, measured in the nigrostriatal pathway at PD diagnosis, may be useful in predicting clinical progression of motor handicap.

## Introduction

Parkinson’s disease (PD) is characterized by the progressive loss of dopaminergic neurons in the nigrostriatal pathway^1,2^. Patients meet the clinical criteria for PD when 60–70% of the neurons in the substantia nigra have degenerated and approximately 80% of the striatal dopamine content has been lost^3,4^. At present, the diagnosis of PD is based primarily on clinical features. Conventional neuroimaging modalities are of proven value in assessing Parkinsonian patients. ^123^I-ioflupane striatal dopamine transporter SPECT (DaTSCAN) has been validated as a tool for the differential diagnosis of PD and non-degenerative tremors, and ^123^I-metaiodobenzylguanidine SPECT and ^18^F-fluorodeoxyglucose PET have been used for the differential diagnosis of PD and atypical Parkinsonism. The very high cost and limited availability of these technologies prevent their widespread and systematic use in routine clinical practice. Moreover, these methods have not been demonstrated to have any prognostic value^5^.

Magnetic resonance imaging (MRI), with different modalities having diverse tissue-specific sensitivities, is useful for investigating brain degeneration to differentiate between PD and other Parkinsonian syndromes. T2* relaxometry and susceptibility-weighted mapping can be used to quantify the iron load and nigral degeneration. Patients with PD have an abnormally low T2 to T2* ratio and, reciprocally, an abnormally high R2 to R2* ratio^6^, while resting state functional connectivity maps describe functional networks; the main finding is that these networks have fewer connections in patients with PD^7^. However, due to the fact that Parkinson's disease’s diagnosis is based on clinical examinations, in addition to the non-availability of all these MR sequences in daily routine and the lack of consensus on the interpretation of images through dedicated software, none of these methods is currently used in clinical practice for diagnostic, therapeutic or prognostic purposes.

Structural imaging, using T1-weighted sequences, has also been investigated and different studies have reported changes in grey matter volume and cortical changes, mainly linked to disease-related cognitive decline^8,9^. The techniques used can be classified into regions-of-interest (ROI)-based approaches with manual labelling, automatic or semi-automatic segmentation, voxel-based whole-brain morphometric analysis using voxel-based morphometry (VBM) or tensor-based morphometry (TBM), and surface or shape-based approaches mainly for cortical thickness analysis. The main results of these studies are inconsistent, however, suggesting that this imaging technique is not sensitive enough and is of little diagnostic value^10^. Conversely, optimized neuromelanin-sensitive T1-weighted scans have revealed stage-dependent substantia nigra signal reduction in PD as a putative marker of neuromelanin loss^11^.

In the present study, we investigated post-processing of T1-weighted images using texture analysis after hypothesizing that the computed features in different key anatomical structures could be clinically meaningful in differentiating between PD patients and controls and show a specific progressive pattern between two critical periods of the disease: diagnosis (early-stage) and severe L-dopa-related complications (late-stage). A number of studies of MRI sequences in patients with Alzheimer’s disease have already found that image texture analysis is more sensitive than conventional atrophy measurements, such ROI volumetry and VBM^12,13^. In a longitudinal study of PD patients with a 2-year follow-up using T2-weighted images, Sikiö et al.^14^, described texture analysis as a quantitative method for detecting structural changes in brain MR images and this was significantly related to clinical scores for PD severity. The Unified Parkinson's Disease Rating Scale (UPDRS) 1 score evaluating the cognition, behaviour and mood, correlated with texture features extracted from the area of posterior corona radiata and from the substantia nigra. UPDRS-II which scores the self-evaluation of the activities of daily living correlated with texture features from the putamen, the dentate nucleus and the thalamus while UPDRS-III score evaluating the motor examination, correlated with features in the area of basilar pons. Li et al.^15^ described the use of quantitative susceptibility-weighted and R2* MR maps to discriminate between patients and healthy controls. More recently, our group has described some texture features as markers of cognitive decline with a better sensitivity than ROI- and VBM-based techniques^16^.

## Results

### Volumetry and morphometry

**Figure 1:**
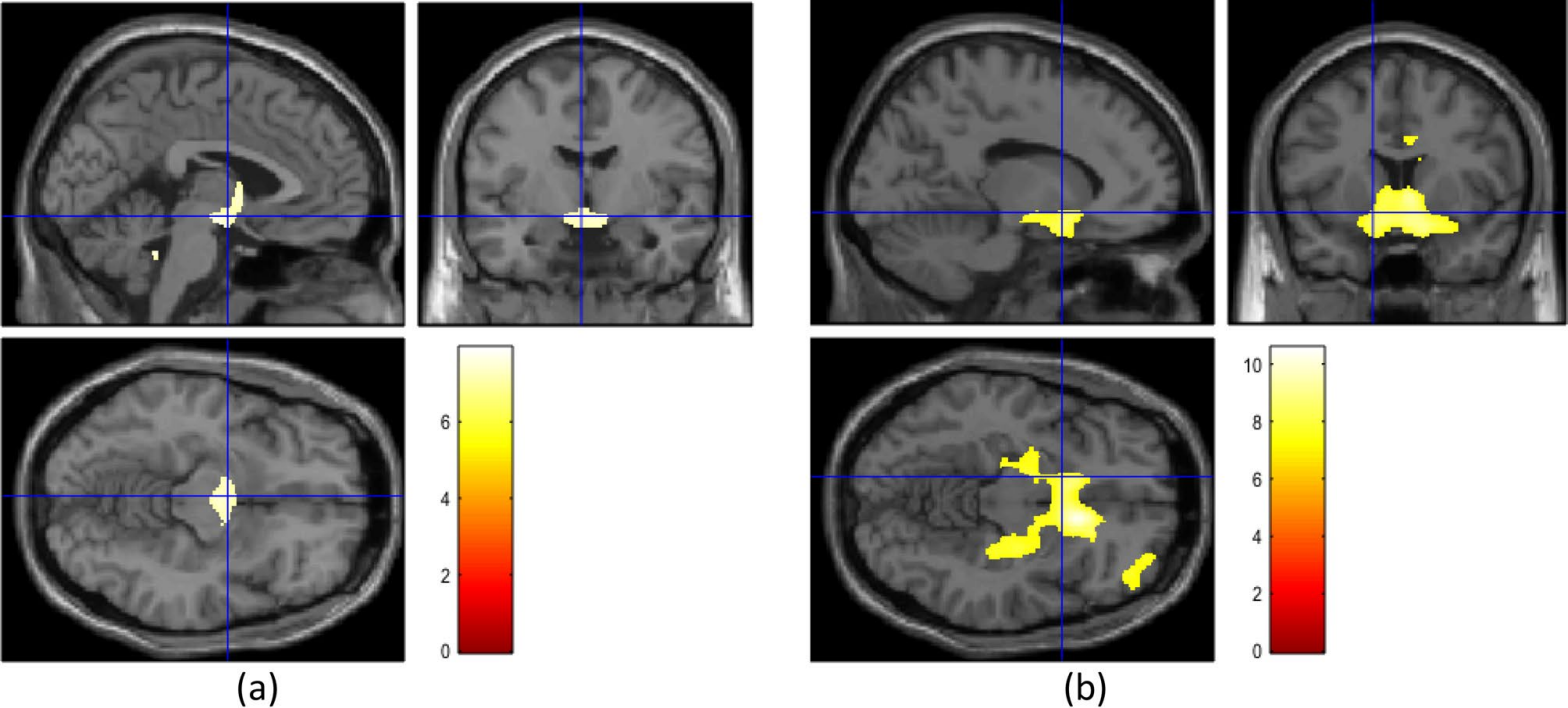
Voxel based morphometry analysis results indicating regions where structural changes between the groups were found using the contrast gray matter in the group 1 > gray matter in the group 2. (**a**) When the healthy group was compared against the early-stage Parkinson’s disease group. (**b**) When the healthy group was compared against the late-stage Parkinson’s disease group. Images created by SPM12, Welcome Trust Centre for NeuroImaging, London, UK; http://fil.ion.ucl.ac.uk/spm/software/spm12/).

There was no significant difference in volume of any of the five brain regions between the two PD groups and the healthy controls. The results of these analyses are shown in Table 1. When compared using the VBM approach, the three groups exhibited significant differences only when the control group was compared with one of the PD patients groups (Fig. 1). These differences were mainly in the deep grey matter structures. No significant difference was found between the two PD patient groups.

**Table 1:** Region of interest-based volumes (expressed in cm^3^) and results of ANOVA for intergroup comparisons after normalization against total intracranial volume.

	Healthy controls (N = 32)	Patients with early-stage PD (N = 39)	Patients with late-stage PD (N = 37)	p
TIV	1193.91 ± 98.50	1184.51 ± 154.55	1155.85 ± 160.15	
SN (right)	0.144 ± 0.018	0.138 ± 0.02	0.130 ± 0.013	0.12
SN (left)	0.142 ± 0.022	0.128 ± 0.016	0.125 ± 0.020	0.08
Putamen (right)	4.51 ± 0.62	4.50 ± 0.62	4.12 ± 0.96	0.11
Putamen (left)	4.57 ± 0.74	4.55 ± 0.58	4.18 ± 0.85	0.10
Thalamus (right)	7.35 ± 0.92	7.48 ± 0.86	7.65 ± 0.99	0.18
Thalamus (left)	7.26 ± 0.84	7.28 ± 1.08	7.26 ± 1.15	0.13
Caudate (right)	3.90 ± 0.50	3.86 ± 0.60	3.82 ± 0.74	0.12
Caudate (left)	3.85 ± 0.46	3.65 ± 0.53	3.60 ± 0.58	0.08
STN (right)	0.112 ± 0.018	0.108 ± 0.019	0.104 ± 0.019	0.09
STN (left)	0.106 ± 0.02	0.103 ± 0.017	0.101 ± 0.01	0.10

*PD* Parkinson’s disease, *TIV* total intracranial volume, *SN* substantia nigra, *STN* sub-thalamic nucleus.

### Texture features

The ANOVA analyses run on the texture features in the five brain structures revealed significant differences between the three groups, with different profiles. For group pair-wise comparisons, significance was considered at p < 0.02 after FDR correction.

**Figure 2:**
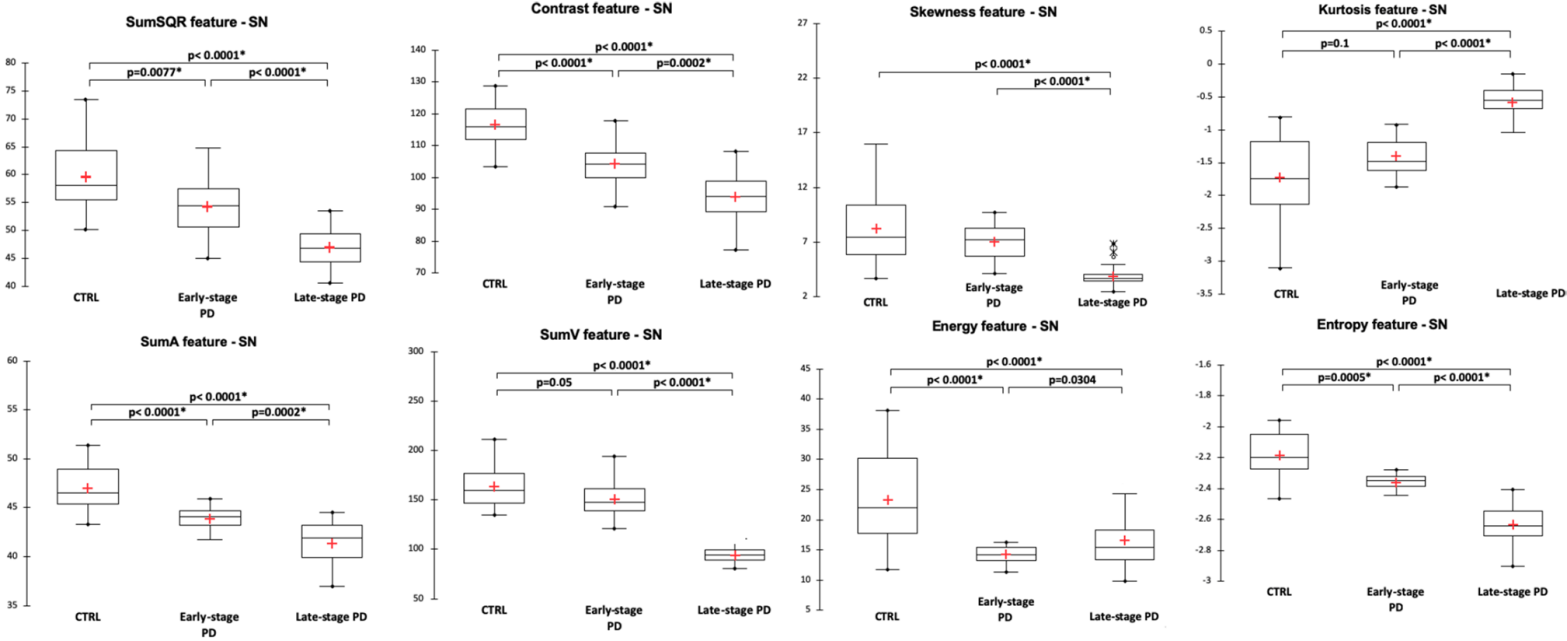
Comparison of texture features in the substantia nigra between healthy controls (CTRL), early-stage PD and late-stage PD patients.

**Figure 3:**
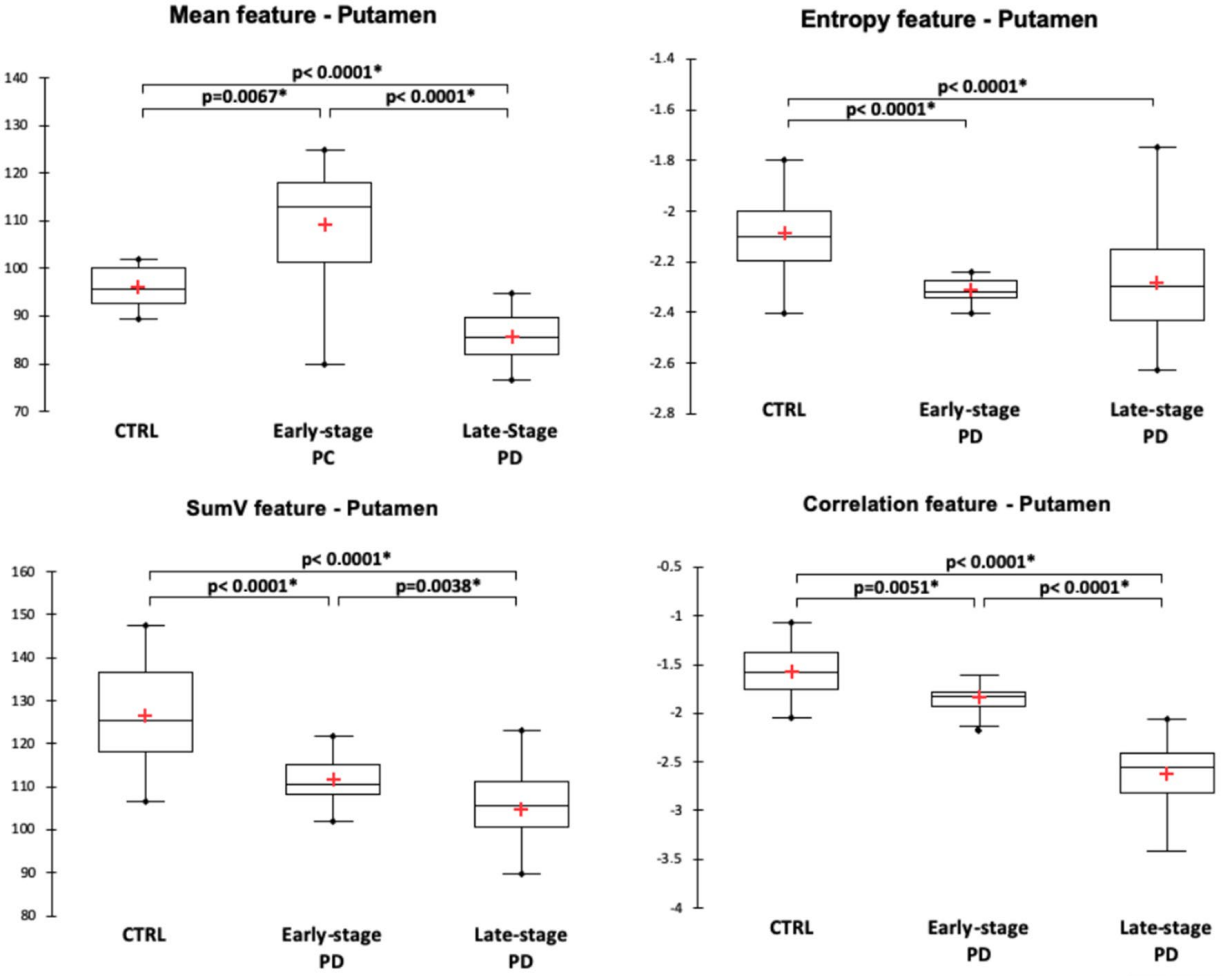
Comparison of texture features in the putamen between healthy controls (CTRL), early-stage PD and late-stage PD patients.

**Figure 4:**
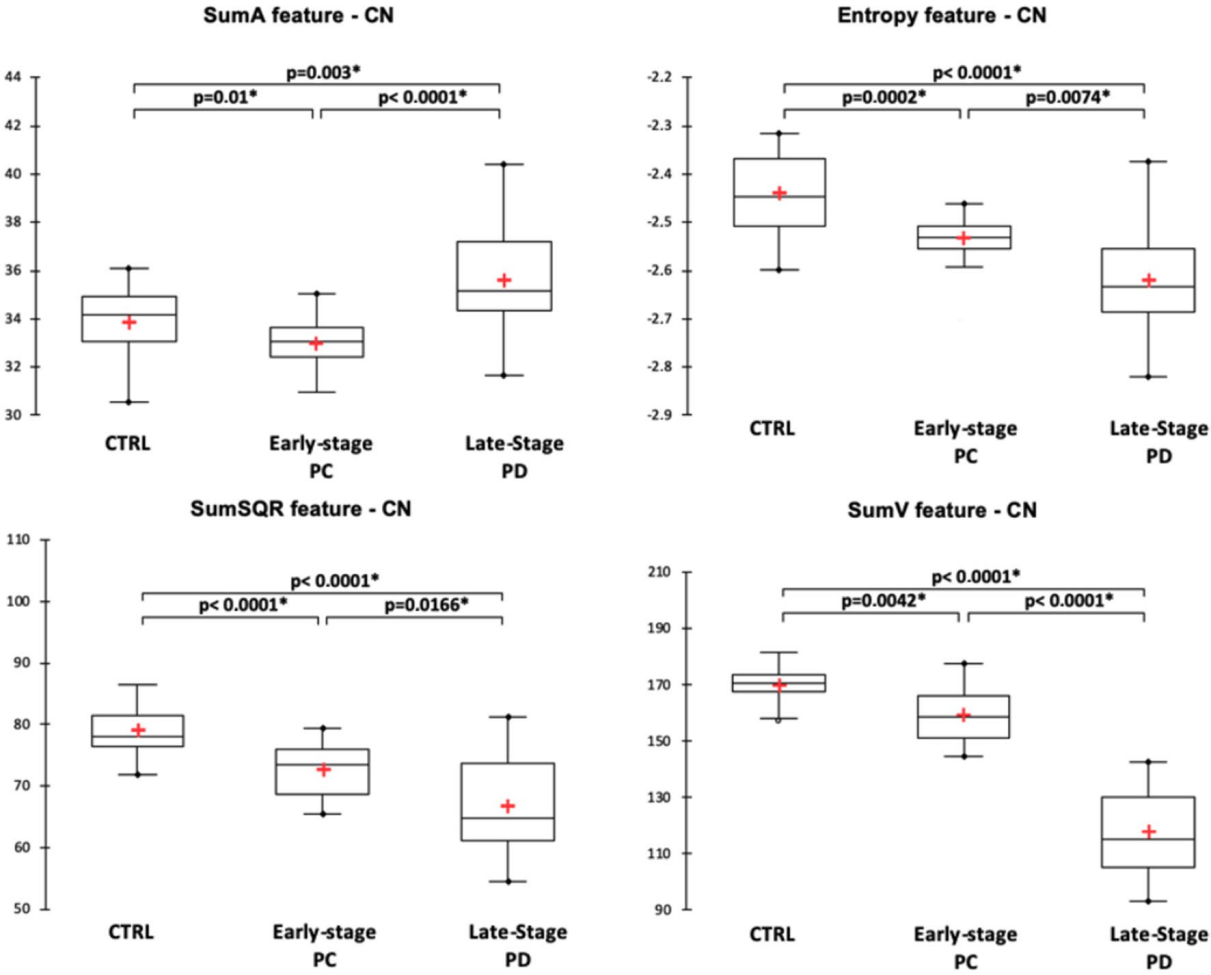
Comparison of texture features in the caudate-nucleus between healthy controls (CTRL), early-stage PD and late-stage PD patients.

**Figure 5:**
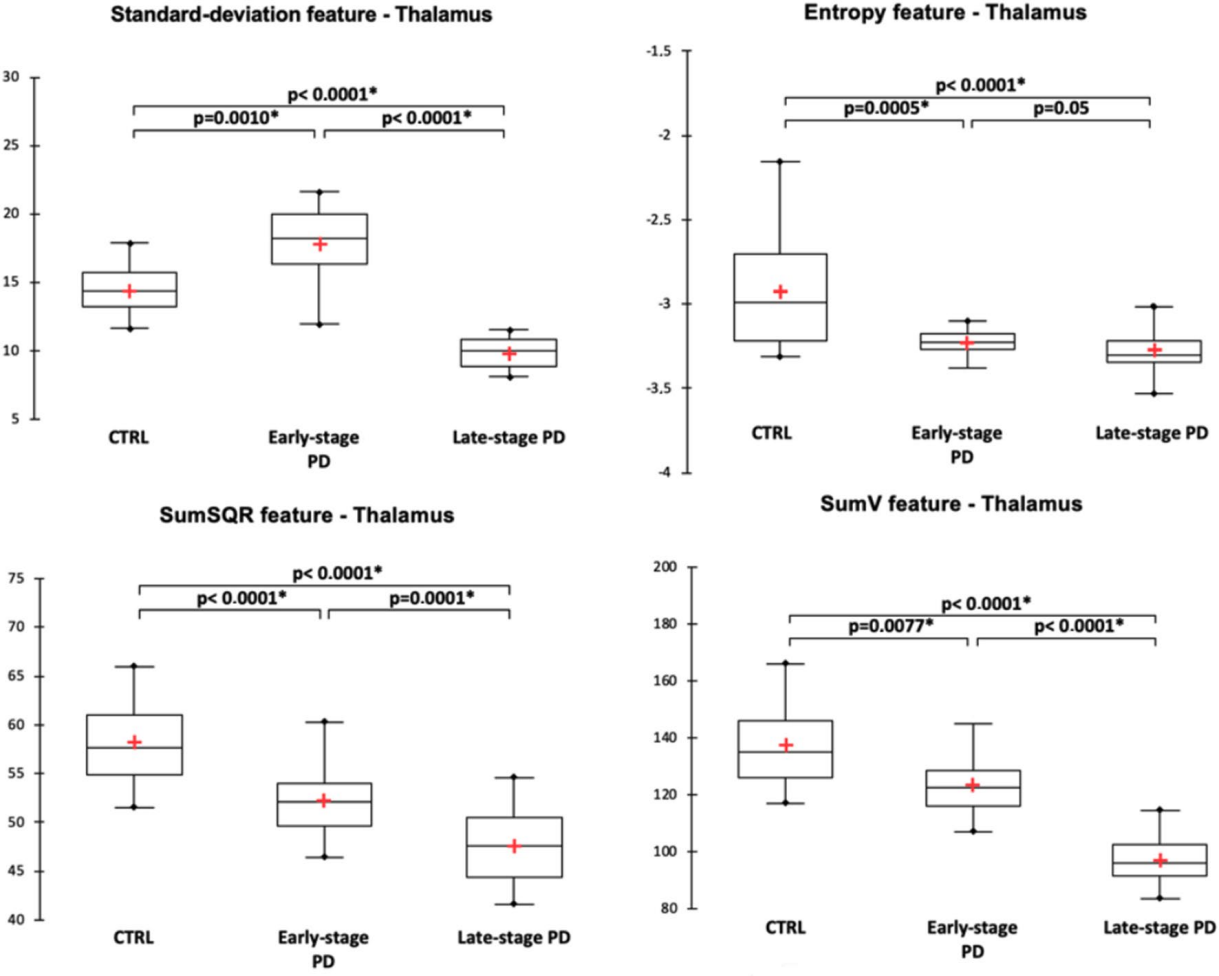
Comparison of texture features in the thalamus between healthy controls (CTRL), early-stage PD and late-stage PD patients.

For the substantia nigra, texture features were significantly different between the three groups and pair-wise post-hoc tests showed that the different features expressed as contrast, entropy, sum variance (sumV) and sum square (sumSQR) were significantly different between the groups. Figure 2 illustrates the distribution of the significantly different texture features. In the striatum, different texture features exhibited pair-wise differences between the three populations. For the putamen (Fig. 3), it was the case for the mean, sumV and correlation features, while for the caudate nucleus (Fig. 4), it was the case for sumA, entropy, sumSQR and sumV. For the thalamus, pair-wise differences were found for the standard-deviation, sumSQR, sumV and entropy features (Fig. 5). Lastly, for sub-thalamic nucleus, this particular structure in terms of volume, two texture features: entropy and sumSQR showed pair-wise differences between the three groups.

### Individual classification using texture features

The regression analysis using the LASSO method showed that three texture features from second-order statistics: entropy, sumSQR and sumV, computed in the five brain regions were the best independent predictors in building a classification model. These features, as it was shown in Figs. 2, 3, 4 and 5, were significantly different between the three populations with a gradual progression from the healthy controls to late-stage PD patients. The discriminatory powers of these texture features between the two PD patient groups were analysed using ROC analysis. The results are summarized in Table 2.

**Table 2:** Receiver operating characteristic (ROC) analysis of the three texture features entropy, sum square (SumSQR) and sum variance (SumV) for discrimination of the two PD patient groups.

	Entropy			SumSQR			SumV		
	AUC	Sp	Se	AUC	Sp	Se	AUC	Sp	Se
Substantia nigra	0.76	0.72	0.73	0.66	0.65	0.70	0.71	0.74	0.73
Putamen	< 0.5			< 0.5			0.62	0.65	0.66
Caudate nucleus	0.63	0.66	0.65	0.55	0.58	0.56	0.64	0.65	0.65
Thalamus	0.54	0.56	0.57	0.69	0.70	0.70	0.79	0.78	0.78
Sub-thalamic nucleus	< 0.5			< 0.5			< 0.5		

*AUC* area under the curve, *Sp* specificity, *Se* sensitivity.

### Texture feature and clinical scores

The correlation analyses with the clinical scores: MDS-UPDRS III and Hoen-Yahr score in off-treatment conditions as well as the MDS-UPDRS total score focused on the same features. After FDR correction, entropy showed significant negative correlations with the three clinical scores (r = − 0.50, p < 0.0001, r = − 0.30, p = 0.01 and r = − 0.45, p = 0.001, respectively) in the substantia nigra. For the putamen, a correlation was found only for the Hoen–Yahr score (r = − 0.30, p = 0.01). For the caudate nucleus, entropy was correlated with the MDS-UPDRS III (r = − 0.41, p = 0.0004) and MDS-UPDRS total (r = − 0.36, p = 0.002) while for the thalamus, it correlated significantly with the three scores (r = − 0.48, p = 0.01, r = − 0.28, p = 0.01 and r = − 0.44, p = 0.002, respectively).

For the feature sumV, significant negative correlations were found with the three clinical scores in the substantia nigra (r = − 0.38, p = 0.002, r = − 0.28, p = 0.01, r = − 0.37, p = 0.002, respectively), caudate nucleus (r = − 0.64, p < 0.0001, r = − 0.49, p < 0.0001, r = − 0.64, p < 0.0001, respectively) and thalamus (r = 0.67, p < 0.0001, r = -0.39, p = 0.001, r = -0.64, p < 0.0001, respectively). For the putamen, this feature correlated with the two MDS-UPDRS scores (r = -0.35, p = 0.003 for MDS-UPDRS III and r = -0.35, p = 0.002 for MDS-UPDRS total).

Finally, for sumSQR, significant correlations with the three clinical scores were found only for the substantia nigra (r = − 0.45, p < 0.0001, r = − 0.27, p = 0.003, r = − 0.40, p = 0.001, respectively). For the caudate nucleus, a significant correlation was found for the MDS-UPDRS III (r = − 0.26, p < 0.015). For the thalamus, correlations were found for the two MDS scores: (r = − 0.45, p < 0.0001 for the MDS-UPDRS III and r = − 0.39, p = 0.001 for the MDS-UPDRS total).

## Discussion

This study investigated T1-weighted MR image texture features that could potentially be used as imaging markers in PD. This imaging sequence is standard in neuroimaging. The method was applied to three populations matched in terms of age and sex. The first population comprised healthy controls while the other two consisted of PD patients at two pivotal stages of the disease: diagnosis (early-PD), when a surrogate biomarker is needed, and late-stage PD with severe L-dopa-related complications, which represents the endpoint for the classical segmental motor handicap of PD. The texture features were computed in different grey matter structures considered as key structures in PD. In addition to the substantia nigra, the primary site of the disease, the putamen and caudate nucleus were also considered as they are directly downstream from the substantia nigra^17^. The thalamus was considered as the master relay station for brain structures, connecting the basal ganglia to the cortical regions, traditionally associated with the disease, and finally, the sub-thalamic nucleus was investigated as it has an important role in the motor system and is of clinical interest as a target in deep brain stimulation. For each structure, 12 texture features from first- and second-order statistics were computed enabling us to obtain a broad description of the grey matter variations inside in each structure.

The structures considered are functionally different in terms of their involvement in PD and its progression at different stages. The substantia nigra and sub-thalamic nucleus have small volumes and may be less suitable for texture analysis. Nevertheless, our results show that some features differed significantly between the three populations. These results confirm the working hypothesis that texture features that quantify grey matter variations can be more sensitive than atrophy measurement methods such as ROI-based volumetry and morphometry. By comparison, in the ROI-based approach, although a global volume diminution can be observed from the healthy group to the late-stage PD group (Table 1), no statistically significant differences were found, while in the VBM approach, differences were observed only when the healthy group was considered against the PD groups (Fig. 1). This approach acts voxel by voxel and consequently can be considered as close to the texture analysis formalism.

The results were more significant in the substantia nigra than in the putamen and to a lesser extent in the caudate nucleus, thalamus and sub-thalamic nucleus. This is congruent with the classical pattern of degeneration predominant in the substantia nigra and putamen at the time of diagnosis and progression in these areas together with the progression of motor handicap^18^. The discriminatory powers of the texture features for the classification of PD patients were also examined using ROC analysis and showed that in most cases the AUC was > 0.5.

Our study also showed that texture features were correlated with classical motor handicap scores in PD. This result suggests that grey matter variations quantified by texture could be clinically meaningful.

By design, texture analysis seems to be less affected by the accuracy of the boundaries of the brain structures than volumetry methods. The most widely used segmentation techniques are still prone to low precision and systematic bias, whereas manual segmentation is time consuming and susceptible to inter-observer variability^19^. In a previous study^20^, we reported the results on the stability and reproducibility of texture features in the hippocampus and entorhinal cortex for discriminating patients with cognitive impairment after a stroke. VBM approaches, that operate on the whole brain, are not reliant on structure definition, but may be hampered by differences in experimental design as the choice of multiple comparison correction techniques lead to differing results^21,22^. Furthermore, in PD, texture features were reported to have higher sensitivity for the detection of slight cognitive slowing^16^.

Our analysis showed that among the 12 texture features considered, three second-order features: entropy, sumV and sumSQR, consistently showed significant differences with a gradual decrease from the healthy control group to late-stage PD. These results are consistent with those of Li et al*.*^15^, where different texture features computed in the substantia nigra on quantitative susceptibility and R2* maps were able to differentiate PD patients from healthy volunteers. These authors identified entropy as one of the most discriminating features, with significantly lower values in the PD patient group. Entropy represents a measure of randomness in the MR signal distribution while sumSQR and sumV reflect signal variation. However, without an anatomo-histological validation study associating a biological signature to each feature, it will remain difficult to have a complete understanding of these texture features.

In-homogeneities and inter-machine variability in T1-weighted signal distribution that can hamper the use of texture analysis were mitigated by the application of normalized grey levels using a N3 algorithm. This processing allowed the standardization of grey level value distributions. Furthermore, the three texture features that appeared as potential markers of PD were obtained from second-order statistics. In contrast to first-order statistic features that are computed directly from the signal values, these features are computed from the co-occurrence matrix making them less sensitive to signal variations.

Finally, it is important to consider the clinical implications of the results of this study. Different studies have attempted to evaluate structural imaging as a tool for the early diagnosis of PD and for differentiating between PD and atypical Parkinsonism. However, despite these efforts, the reality is that there is not enough statistical separation of single-structure measurements between PD and non-PD subjects to be of clinical use^10^. The current trends suggest considering a pattern of atrophy across several structures^23^. The approach proposed here is in line with these trends; the combination of different features, computed in different structures of interest, is more suitable for machine learning and prediction models to make individualized patient decisions. A multi-parametric and multi-modality solution, involving different MR sequences (T1 and R2*) and/or SPECT images may enhance the predictive ability of the model^24,25^.

## Methods

### Study population

The PD population was enrolled consecutively, from the movement disorders department of Lille university hospital, following the inclusion and non-inclusion criteria, described below, of early PD at the time of diagnosis and advanced PD at the time of motor fluctuations. In a second step, healthy controls were selected to match to the age and sex ratio of the PD patients. The healthy controls were recruited among spouses and caregivers who did not have neurological pathologies or other serious conditions (progressive inflammatory or cancerous pathology). There were no secondary exclusions after inclusions for medical (claustrophobia, incidentaloma, etc.) or technical (artifacts, etc.) reasons.

Thirty-nine de novo diagnosed patients before any symptomatic treatment, mean disease duration 0.45 year, and median Hoehn–Yahr stage 2 (min = 0 and max = 3) and late-stage patients with severe motor fluctuations and candidate for second-line treatments (i.e. apomorphine pump, deep brain stimulation, DUODOPA), mean disease duration 8.45 years, and median Hoehn–Yahr stage 2.5 (min = 1 and max = 5) were included. All patients met the Movement Disorders Society (MDS) clinical criteria for the diagnosis of PD^26^. Patients with severe cognitive impairment or dementia [Montreal Cognitive Assessment score < 22 and as defined by the Diagnostic and Statistical Manual of Mental Disorders, Fourth Edition (DSM-IV) criteria], patients with psychiatric disorders (psychosis, hallucinations, compulsive disorders, substance addiction, bipolar disorder, severe depression, according to the DSM-IV), as assessed in a semi-structured interview with a psychiatrist, and patients with severe brain atrophy or abnormal MRI results for any reason other than PD were excluded from the study.

The demographic description of the population and clinical characteristics of the patients are summarized in Table 3.

**Table 3:** Demographic and clinical characteristics of the three study populations.

	Healthy controls (N = 32)	Patients with early-stage PD (N = 39)	Patients with late-stage PD (N = 37)	*p*
Sex (M/F)	18/14	22/17	22/15	0.30*
Age (years)	61.6 ± 10.8	63 ± 10.4	60.75 ± 9.6	0.40**
Disease duration (years)	–	0.45 ± 0.46	8.5 ± 3.9	< 0.0001^#^
Hoehn–Yahr score (OFF)	–	2 (0–3)	2.5 (1–5)	< 0.0001^#^
MDS-UPDRS 1	–	8.25 ± 5.70	10.2 ± 4.9	0.02^#^
MDS-UPDRS 2	–	5.66 ± 4.09	17.8 ± 7.16	< 0.0001^#^
MDS-UPDRS 3 (OFF)	–	17.15 ± 7.40	45.42 ± 16.38	< 0.0001^#^
MDS-UPDRS 4	–	0	9.0 ± 3.7	–
MDS-UPDRS total	–	32.05 ± 12.94	78.86 ± 24.44	< 0.0001^#^

*M* male, *F* female, *PD* Parkinson’s disease, *MDS-UPDRS* Movement Disorders Society-Unified Parkinson's Disease Rating Scale, *OFF* off-treatment.

*Pearson’s Chi-squared test. **ANOVA. ^#^Mann–Whitney test.

The study was approved by the institutional review board (Comité de Protection des Personnes (CPP) Nord Ouest IV, Lille, France; study reference: 2013-A00193-42). All participants provided their written, informed consent before participation. The study complied strictly with the methods, guidelines and regulations described in the approved protocol.

### Image acquisition and structures of interest

All patients and controls were scanned using 3 T MRI systems (PHILIPS Healthcare, Best, Netherlands) with an 8-channel sensitivity encoding (SENSE) head coil. High-resolution 3D T1-weighted images were acquired in the sagittal plane with 1 mm^2^ isotropic pixel size, repetition time = 7.2 ms, echo time = 3.3 ms, flip angle = 9°, field of view = 240 × 256 mm^2^; acquisition matrix = 256 × 256; slice thickness = 1 mm and 176 continuous slices.

A loss of nigral dopaminergic neurons is strongly correlated with the motor impairments that characterize PD^27^. However, regions of cell loss also include the caudate nucleus^28^, thalamus^29^ and putamen^30^. For this study, we considered the following deep grey matter structures: substantia nigra, putamen, thalamus, caudate nucleus and sub-thalamic nucleus. For the putamen, thalamus and caudate nucleus, the left and right parts of each hemisphere were extracted from the images using Freesurfer. The substantia nigra and sub-thalamic nucleus were segmented using in-house software that implements an atlas-based approach for the substantia nigra atlas^31^ and the sub-thalamic nucleus atlas^32^. The results were checked visually and corrected as appropriate.

### Volumetry and morphometry

Classical MRI approaches based on brain atrophy measurement were investigated to test for group differences. ROI based volumetry and VBM were used. For the first, bilateral volumes of each structure, described above, were estimated from the segmentation data. Normalization was done by dividing each individual volume by the intracranial volume (ICV)^33^. The ICV was estimated as a part of the static FreeSurfer pipeline using a method described in Buckner et al*.*^34^.

For the VBM method, images were processed using the SPM12 DARTEL toolbox (Welcome Trust Centre for NeuroImaging, London, UK; http://fil.ion.ucl.ac.uk/spm/software/spm12/) with default settings^35^.

### Texture features

**Figure 6:**
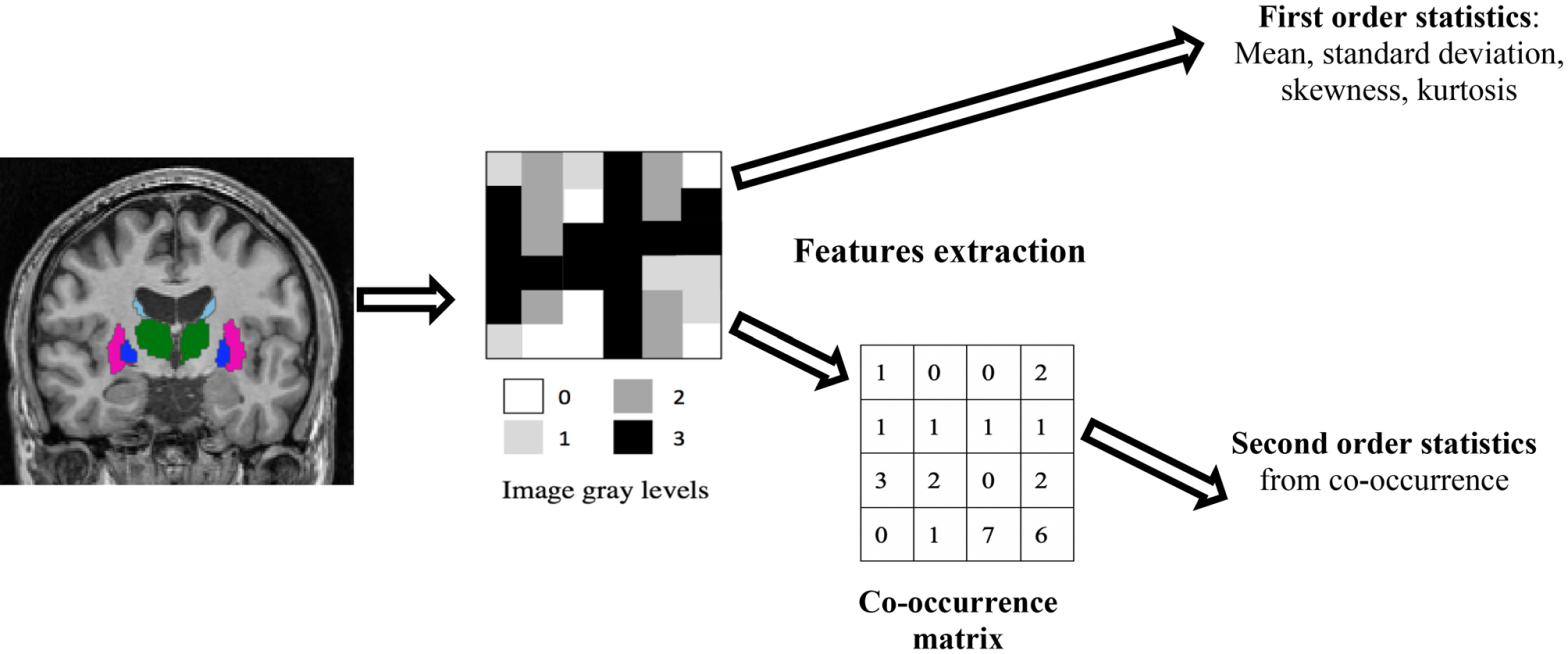
Texture features extraction scheme. For each brain structure represented as a region of interest segmented on the T1w MR images, gray levels variation is captured without taking into account voxels neighbouring using first order statistics and by considering the neighbourhood by converting the gray levels into a co-occurrence matrix and then extracting the second order statistics.

Image texture can be described using different features. In this study, under the assumption that the neural loss induced by the disease progression affects the signal distribution in the region of interest, texture was captured using features from the first order statistics and in order to take into account the neighbourhood in gray levels variation, second order statistics, derived from the co-occurrence matrices were used (Fig. 6). In total, twelve texture features were computed: four from first-order statistics and six from second-order statistics, used in our previous investigation^16^. The first-order parameters included mean grey level, standard deviation (SD) of grey levels, kurtosis (a measure of whether intensities are heavy-tailed or light-tailed, relative to a normal distribution) and skewness (a measure of a lack of symmetry of the signal intensity). The second-order features (also known as Haralick texture features) quantify the relationships between pairs of neighbouring voxels in the image. The features were derived from the grey level co-occurrence matrix (GLCM); a spatial relationship was defined as the relative direction in a given direction d. In this study, the GLCM matrix was estimated by considering four directions (θ = 0°, 45°, 90° and 135°) and a distance d = 1. Using this matrix, the following features were computed: homogeneity, contrast, entropy, correlation, variance, sum average, sum variation and inverse different moment (IDM). All the features and their computation are described in Table 4.

**Table 4:** Texture features considered, together with their significance, equations and models.

Feature	Description	Equation
First-order statistics		
Mean	Mean image grey level values	
SD	Standard deviation of grey level values	
Kurtosis (Kurt)	Kurtosis is a measure of whether the data are heavy-tailed or light-tailed, relative to a normal distribution. Positive kurtosis indicates a peaked distribution, while negative kurtosis indicates a flat distribution	$\mathrm{Kurt}=\mathrm{E}\left[{\left(\frac{\mathrm{X}-\mathrm{Mean}}{\mathrm{SD}}\right)}^{4}\right]$
Skewness (Skew)	Skewness quantifies the lack of symmetry: it is zero for a symmetric distribution and negative for left-skewed data	$\mathrm{Skew}=\mathrm{E}\left[{\left(\frac{\mathrm{X}-\mathrm{Mean}}{\mathrm{SD}}\right)}^{3}\right]$
Second-order statistics		
Homogeneity contrast (Cont)	Uniformity of texture intensity (a measure of the closeness of the distribution of elements in the co-occurrence matrix) Contrast represents the degree to which the texture intensity levels differ between voxels (i.e. local intensity variations). It will favour contributions from p(i,j) away from the diagonal	$\mathrm{Homogeneity}= \sum_{\mathrm{i}=0}^{\mathrm{G}-1}\sum_{\mathrm{j}=0}^{\mathrm{G}-1}{(\mathrm{P}(\mathrm{i},\mathrm{j}))}^{2}$ $\mathrm{Contrast}= \sum_{\mathrm{i}=0}^{\mathrm{G}-1}\sum_{\mathrm{j}=0}^{\mathrm{G}-1}{(\mathrm{i}-\mathrm{j})}^{2}\cdot \mathrm{P}(\mathrm{i},\mathrm{j})$
Entropy (Ent)	Entropy represents the degree of uncertainty (a measure of randomness)	$\mathrm{Entropy}=-{\sum }_{\mathrm{i}=0}^{\mathrm{G}-1}{\sum }_{\mathrm{j}=0}^{\mathrm{G}-1}\mathrm{P}\left(\mathrm{i},\mathrm{j}\right)\cdot \mathrm{log}(\mathrm{P}(\mathrm{i},\mathrm{j}))$
Correlation (Corr)	Correlation represents the degree of mutual dependency between pixels	$\mathrm{Correlation}={\sum }_{\mathrm{i}=0}^{\mathrm{G}-1}{\sum }_{\mathrm{j}=0}^{\mathrm{G}-1}\frac{\left\{\mathrm{i}\cdot \mathrm{j}\right\}\cdot \mathrm{P}\left(\mathrm{i},\mathrm{j}\right)-\left\{{\upmu }_{\mathrm{x}}\cdot {\upmu }_{\mathrm{y}}\right\}}{{\upsigma }_{\mathrm{x}}\cdot {\upsigma }_{\mathrm{y}}}$
Sum of squares (SumSqr)	Sum of squares also called variance gives a high weighting to elements that differ from the average value	$\mathrm{SumSQR}=\sum_{\mathrm{i}=0}^{\mathrm{G}-1}\sum_{\mathrm{j}=1}^{\mathrm{G}-1}(\mathrm{i}-\upmu )^{2}\mathrm{P}(\mathrm{i},\mathrm{j})$
Sum average (SumA) Sum variation (SumV)	Sum average measures the relationship between occurrences of pairs with lower intensity values and occurrences of pairs with higher intensity values. Quantifies brightness Sum variation represents the global variation in the sum of the grey-levels of voxel-pairs distribution	$\mathrm{SumAvg}=\sum_{\mathrm{I}=1}^{2\mathrm{G}}\mathrm{i}{\cdot \mathrm{P}}_{\mathrm{x}+\mathrm{y}}(\mathrm{j})$ $\mathrm{SumV}=\sum_{\mathrm{I}=1}^{2\mathrm{G}}{\left(\mathrm{i}-\mathrm{SumAvg}\right)}^{2}{\cdot \mathrm{P}}_{\mathrm{x}+\mathrm{y}}(\mathrm{i})$
Inverse difference moment (IDM)	The IDM corresponds to small contributions from in-homogeneous areas (i / = j). The value is low for in-homogeneous images and relatively high for homogeneous images	$\mathrm{IDM}= \sum_{\mathrm{i}=0}^{\mathrm{G}-1}\frac{\mathrm{P}\left(\mathrm{i},\mathrm{j}\right)}{1+{\left(i-j\right)}^{2}}$

*G* number of grey levels used, *I* intensity value of a neighbour voxel, *j* intensity value of a reference pixel, *P(i,j)* probability of the appearance of the (i,j) pair in the co-occurrence matrix, $\mu \mathrm{ m}$ ean value of P, *P*_*x*_* and P*_*y*_ marginal probabilities, ${\sigma }_{x}.{\sigma }_{y}$ SD of P_x_ and P_y_, respectively.

$${\mathrm{P}}_{\mathrm{x}+\mathrm{y}}=\sum_{\mathrm{i}=0}^{\mathrm{G}-1}\sum_{\mathrm{j}=1}^{\mathrm{G}-1}\mathrm{P}(\mathrm{i},\mathrm{j})$$, i + j = k for k = 0,1….G−1.

For each brain structure and each feature, calculation was done for the right and left sides and then averaged.

Texture features may be affected by MR signal in-homogeneities and inter-machine variability in T1-weighted sequences making their reproducibility questionable. In order to ensure reproducibility, the images were corrected for field bias and in-homogeneities using the nonparametric non-uniform intensity normalization algorithm (N3). This processing allowed standardization of grey level value distributions (FreeSurfer software package (version 6.0)^36^.

### Statistical analysis

Texture features and ROI-based volumes were compared between the three groups using ANOVA with significance fixed at p < 0.05. If appropriate, t tests with false rate discovery (FDR) correction were then run for pair-wise comparisons. For the VBM, t tests were performed to identify differences in whole brain grey matter volume. Clusters were considered significant with a threshold in terms of size fixed to 10 voxels and after controlling for family-wise errors rate using FDR.

Regression analysis using the least absolute shrinkage and selection operator (LASSO) method was run on the statistically significant texture features to select the best independent predictors. The LASSO analysis was run by considering the three groups. The individual classification capabilities in separating the two PD groups of the selected features were subsequently measured using area under curve (AUC) analysis.

Correlations between the texture features and classical clinical motor scores (MDS-UPDRS III, Hoehn–Yahr score and MDS-UPDRS total) were investigated using Spearman’s correlation coefficient with significance fixed at p < 0.05 and corrected using FDR.

Texture features were computed using in-house software and the XLSTAT software plug-in (AddinSoft, www.xlstat.com) was used to perform all statistical tests. The code package as well as all the data used in this study are available for download.
